# Volatile organic compounds of Thai honeys produced from several floral sources by different honey bee species

**DOI:** 10.1371/journal.pone.0172099

**Published:** 2017-02-13

**Authors:** Praetinee Pattamayutanon, Sergio Angeli, Prodpran Thakeow, John Abraham, Terd Disayathanoowat, Panuwan Chantawannakul

**Affiliations:** 1 Department of Biology, Faculty of Science, Chiang Mai University, Chiang Mai, Thailand; 2 Faculty of Science and Technology, Free University of Bozen-Bolzano, Piazza Università, Bolzano, Italy; 3 Division of Product Development Technology, Faculty of Agro-Industry, Chiang Mai University, Chiang Mai, Thailand; University of North Carolina at Greensboro, UNITED STATES

## Abstract

The volatile organic compounds (VOCs) of four monofloral and one multifloral of Thai honeys produced by *Apis cerana*, *Apis dorsata* and *Apis mellifera* were analyzed by headspace solid-phase microextraction (HS-SPME) followed by gas chromatography and mass spectrometry (GC-MS). The floral sources were longan, sunflower, coffee, wild flowers (wild) and lychee. Honey originating from longan had more VOCs than all other floral sources. Sunflower honey had the least numbers of VOCs. *cis*-Linalool oxide, *trans*-linalool oxide, ho-trienol, and furan-2,5-dicarbaldehyde were present in all the honeys studied, independent of their floral origin. Interestingly, 2-phenylacetaldehyde was detected in all honey sample except longan honey produced by *A*. *cerana*. Thirty-two VOCs were identified as possible floral markers. After validating differences in honey volatiles from different floral sources and honeybee species, the results suggest that differences in quality and quantity of honey volatiles are influenced by both floral source and honeybee species. The group of honey volatiles detected from *A*. *cerana* was completely different from those of *A*. *mellifera* and *A*. *dorsata*. VOCs could therefore be applied as chemical markers of honeys and may reflect preferences of shared floral sources amongst different honeybee species.

## Introduction

Honey is a well-known natural product derived from honeybees. Worker honeybees collect nectar from flower blossom and store them in a honey sack before they return to the hive. The nectar is then mixed with enzymes from bees resulting in the breakdown of complex sugars in the nectar to simple sugars such as glucose and fructose [1]. Besides sugar content, honey contains proteins, lipids and vitamins [2]. Honey may have aromatic compounds at very low concentrations in the form of volatile mixtures. Each volatile compound in the mixture may contribute a different aroma, taste and function leading to the uniqueness of honey. Volatile organic compounds (VOCs) contributing to the aroma of honey vary in quality and quantity because of different nectar sources, microbes in honey, transformation of plant compounds by bees, honey processing, and condition of storage [3, 4].

Numerous organic compounds have been detected and identified as characteristic compounds of honey [5–7]. Three well-known principal categories of honey volatiles are terpenes and their derivatives, norisoprenoids, and benzene derivatives [8]. Terpenes and their derivatives are known as important compounds that provide flavor, odor and biomedical properties in honey [9, 10]. Norisoprenoids have very low olfactory perception thresholds which strongly influence honey odor [11]. Aside the impact of norisoprenoids on honey odor, some of them are known to be anticarcinogenic [12]. Norisopreniods are products of Carotenoids [13, 14]. Furthermore, benzene derivatives can be used as pollution-monitor volatiles [15], although they have been found to be the main antibacterial volatile in New Zealand honeys [16]. All these categories of compounds have previously been detected in Thai honeys [17] in which terpenes were found to be the most abundant VOCs in both wild and coffee honeys. Although, there have been previous studies on honeys produced by *Apis mellifera*, information on VOCs from honey derived from other honeybee species are still lacking. In Northern Thailand, three species of honeybees (*Apis mellfiera*, *Apis cerana* and *Apis dorsata*) are known to produce honeys for human consumption. To bridge the information gap on honeys produced by other bee species, this present study aimed at determining the VOCs released by Thai honeys produced from different floral sources and three different honey species. Knowledge from this could inform the characteristics of honeys and subsequently used to distinguish honeys from different floral sources and honey bee species.

## Materials and methods

### Honey samples

Honeys produced from five different floral sources by three species of honey bees were obtained from retailers (processed honey) and beekeepers (unprocessed honey) in different provinces of Thailand (Chiang Mai, Chiang Rai, Lumphun, Nan, and Lopburi). The honey samples and their bee producers were *Apis mellifera* (longan honey, wild honey, lychee honey and sunflower honey), *Apis cerana* (longan honey, wild honey and coffee honey) and *Apis dorsata* (wild honey). All samples were kept in a dark room at 23–25°C until volatile samples were collected from them by solid-phase microextraction (SPME).

### Analysis of volatile organic compounds

#### Sample preparation

For each of the honey samples from different floral sources, 2.5 g of honey was weighed into a 20 ml screw-top glass vial with PTFE/silicone lid. As internal standard, 5 μl of 1 mg/ml benzophenone (Sigma-Aldrich, Milan, Italy) in methanol (Sigma-Aldrich) was used, gently putting it on the inner wall of the vial.

#### Solid-phase microextraction of volatile samples

The volatile samples were analyzed by SPME where vials containing the honey samples and the internal standard were conditioned at 45°C in the agitator of the GC set-up for 15 minutes before SPME extraction. A 2 cm 50/30 μm divinylbenzene/carboxen/polymethylsiloxane (57299-U) SPME fiber was put into the vial to expose it to the headspace of the honey samples. The volatile samples were allowed 40 minutes to be adsorbed onto the SPME fiber at 45°C before GC injection. At injection into the GC, thermal desorption of volatile organic compounds from the SPME fiber lasted 5 minutes.

#### Gas chromatography—mass spectrometry

GC-MS analysis was done by using a GC (7890A, Agilent Technologies, Santa Clara, USA) coupled with an MS (5975C Network, Agilent Technologies). The GC had an HP 5MS column (non-polar column, Agilent Technologies), 30 m x 0.25 mm internal diameter (i.d.) and 0.25 μm film thickness. The carrier gas was helium flowing at a rate of 1.2 mL/ minute. The initial oven temperature was 40°C held for 3 minutes. It was raised to 200°C at 3°C/minute and then 16°C/minute until it reached 240°C and held at this temperature for 1.2 minutes. Mass spectra were recorded in a scanning range of 24–360 m/z.

Enhanced ChemStation (ver. E.02.02.1431, Agilent Technologies) was used for identification by comparing the mass spectra of VOCs with those in the database of NIST 11(Gaithersburg, MD, USA) and Wiley 7N (John Wiley, NY, USA). The linear retention indices (LRI) of the VOCs were calculated using the retention times of n-alkane series from C9 to C20 as reference compounds [18]. The calculated LRI were then compared to those in NIST webbook. Each peak area of characterized compound was compared to peak area of the internal standard (5 μL of 1 mg/mL benzophenone), the amounts of detected VOCs were expressed as relative amount (%) ± standard error.

#### Statistical analysis

The relative amounts of detected VOCs in the honey samples were analyzed for normality (Shapiro-Wilk test), Non-metric multidimensional scaling (NMDS) and grouping plot illustrations were generated using Paleontological statistics; PAST version 2. The normally distributed data were analyzed by one-way ANOVA (SPSS v.17.0; IBM, Chicago, IL, USA). Mann-Whitney tests (SPSS) were applied to data, which were not normally distributed. Significant levels were set at α = 0.05.

## Results

**Fig 1:**
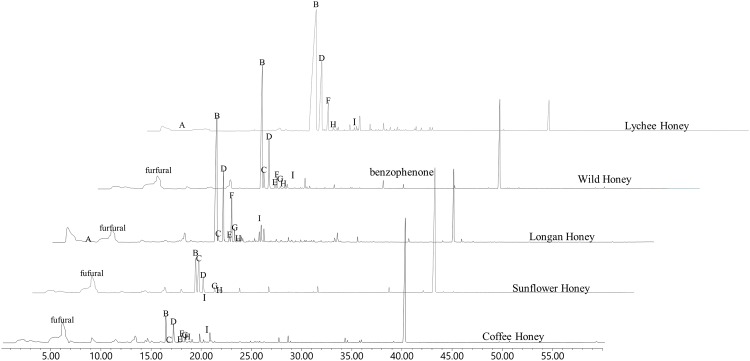
Representative chromatograms of Thai honeys on HP-5MS column. A: isoamyl alcohol. B: c*is*-linalool oxide. C: 2,5-furandicarboxaldehyde. D: *trans*-linalool oxide. E: linalool. F: ho-trienol. G: benzyl ethanol. H: isophorone. I: epoxylinalool.

A total of 62 compounds were identified from the headspace volatiles of the honey samples studied. Of the total number of compounds, 48 were present in longan honey, 41 in wild honey, 21 in lychee honey, 23 in coffee honey and 8 in sunflower honey (Table 1). Moreover, the identified VOCs belonged to 7 chemical classes namely alcohols, aldehydes, ketones, esters, acids, hydrocarbons, and terpenes. Terpenes were the most abundant compounds among all the volatiles in all the samples. Three volatiles namely *cis*-linalool oxide (18.9–1,043.5%), furan-2,5-dicarbaldehyde (1.2–45.9%) and *trans-*linalool oxide (8.2–29.5%) were present in all honey samples (Fig 1). Some volatiles were absent in particular honeys, e.g. 2-phenylacetaldehyde, isophorone and methyl nonanoate were absent from longan honey from *A*. *cerana* while linalool, ho-trienol, benzyl ethanol, isophorone and epoxylinalool isomer II were absent from sunflower honey from *A*. *mellifera*. However, significantly high amounts of *cis*- and *trans*-linalool oxide were found in lychee (*A*. *mellifera*) and longan honeys (*A*. *mellifera* and *A*. *cerana*) than that found in the other honeys. Interestingly, 2-phenylacetaldehyde described as honey, sweet, and floral odor was detected in all honey sample except longan honey produced by *A*. *cerana*. When comparing the numbers of the identified volatile compounds, it was found that longan honey produced by *A*. *mellifera* had the highest amount of volatiles (48 VOCs) while, sunflower honey produced by *A*. *mellifera* has the least amount of volatiles (8 VOCs). The volatiles of longan honey produced by *A*. *mellifera* were double of ones of *A*. *cerana*, 48 and 22, respectively. In addition, the quantities of each volatile compound of longan honey produced by *A*. *mellifera* were generally more than the ones produced by *A*. *cerana*. When considering wild honeys, it was found that the ones produced by *A*. *dorsata* (31 VOCs) contained the highest number of volatile, followed by *A*. *mellifera* (26 VOCs) and *A*. *cerana* (14 VOCs), respectively.

**Table 1:** Relative amounts (%) ± standard error of Thai honeys volatile organic compounds as compared to the internal standard (benzophenone).

No.	LRI	VOCs*	Longan		Wild			Lychee	Coffee	Sunflower
			*A*. *mellifera* (n = 17)	*A*. *cerana* (n = 5)	*A*. *dorsata* (n = 8)	*A*. *mellifera* (n = 9)	*A*. *cerana* (n = 4)	*A*. *mellifera* (n = 4)	*A*. *cerana* (n = 5)	*A*. *mellifera* (n = 3)
**1**	<800	3-methylbutan-1-ol [isoamyl alcohol]	24.39 ± 8.55	17.49 ± 5.90	30.79 ± 15.85	n.d.	n.d.	n.d.	n.d.	n.d.
**2**	830	furan-2-carbaldehyde [furfural]	84.25 ± 21.24	n.d.	60.67 ± 30.93	60.27 ± 14.99	n.d.	24.59 ± 14.20	130.60 ± 55.91	109.92 ± 55.37
**3**	851	2-furanmethanol	n.d.	n.d.	n.d.	n.d.	n.d.	n.d.	12.32 ± 2.54	n.d.
**4**	910	butyryl lactone	n.d.	n.d.	n.d.	n.d.	n.d.	n.d.	21.24 ± 7.76	n.d.
**5**	925	methyl caproate	n.d.	n.d.	1.34 ± 0.91	n.d.	n.d.	n.d.	n.d.	n.d.
**6**	955	benzaldehyde	n.d.	n.d.	2.01 ± 1.32	n.d.	n.d.	n.d.	n.d.	n.d.
**7**	961	5-methylfurfural	n.d.	n.d.	4.62 ± 3.67	n.d.	n.d.	n.d.	12.02 ± 4.74	n.d.
**8**	1002	2-methyl-5-prop-1-en-2-ylcyclohexa-1,3-diene [1,5,8-*p*-menthatriene]	3.46 ± 2.37	n.d.	n.d.	n.d.	n.d.	n.d.	n.d.	n.d.
**9**	1019	1,8-nonadiyne	4.64 ± 1.39	n.d.	n.d.	0.51 ± 0.34	n.d.	n.d.	n.d.	n.d.
**10**	1028	ethyl heptanoate	0.95 ± 0.53	n.d.	n.d.	n.d.	n.d.	n.d.	n.d.	n.d.
**11**	1033	phenylmethanol	n.d.	n.d.	n.d.	n.d.	n.d.	n.d.	10.82 ± 2.64	n.d.
**12**	1041	**2-phenylacetaldehyde**	0.42 ± 0.28	n.d.	5.94 ± 3.06	2.91 ± 0.91	5.18 ± 1.03	1.26 ± 0.74	3.86 ± 1.65	3.51 ± 2.25
**13**	1070	**2-(5-ethenyl-5-methyloxolan-2-yl)propan-2-ol [*cis*-linalool oxide]**	437.57 ± 87.28 ab	377.40 ± 51.77 ab	190.54 ± 58.04 b	177.70 ± 64.95 b	18.91 ± 6.14 b	1043.49 ± 652.53 a	54.90 ± 13.14 b	32.29 ± 18.62 b
**14**	1077	**furan-2,5-dicarbaldehyde**	24.11 ± 10.61 a	1.22 ± 0.76 b	45.92 ± 23.73 a	23.26 ± 6.95 a	1.62 ± 0.95 b	7.16 ± 6.38 b	3.09 ± 1.77 b	33.50 ± 17.88 a
**15**	1089	**2-(5-ethenyl-5-methyloxolan-2-yl)propan-2-ol [*trans*-linalool oxide]**	112.71 ± 22.57 ab	96.37 ± 11.96 ab	80.30 ± 33.91 ab	50.53 ± 17.28 b	8.21 ± 2.32 b	295.47 ± 170.70 a	38.47 ± 8.45 b	11.29 ± 6.52 b
**16**	1100	**3,7-dimethylocta-1,6-dien-3-ol [linalool]**	16.73 ± 3.17 a	6.85 ± 1.27 b	2.74 ± 1.40 b	1.42 ± 0.65 c	0.84 ± 0.50 c	3.97 ± 1.95 b	3.09 ± 1.19 b	n.d.
**17**	1104	**(5E)-3,7-dimethylocta-1,5,7-trien-3-ol [ho-trienol]**	93.57 ± 13.37 a	20.90 ± 6.98 b	11.35 ± 5.65 c	16.54 ± 4.69 c	2.71 ± 1.07 c	72.37 ± 43.08 b	21.24 ± 10.17 b	n.d.
**18**	1111	**benzyl ethanol**	18.32 ± 3.03	5.23 ± 0.87	18.62 ± 7.20	3.73 ± 1.08	4.29 ± 2.02	7.27 ± 6.14	10.65 ± 2.27	n.d.
**19**	1117	**3,5,5-trimethyl-2-cyclohexene-1-one [isophorone]**	3.44 ± 0.76	n.d.	1.83 ± 0.98	6.17 ± 2.28	1.36 ± 0.81	10.42 ± 4.84	1.65 ± 1.07	n.d.
**20**	1126	methyl octanoate	1.22 ± 0.70	n.d.	n.d.	4.25 ± 1.72	n.d.	n.d.	n.d.	n.d.
**21**	1128	(3E,5E)-2,6-dimethylocta-1,3,5,7-tetraene [cosmene]	7.54 ± 1.10	n.d.	n.d.	n.d.	n.d.	7.66 ± 3.14	n.d.	n.d.
**22**	1142	4-oxoisophorone	3.23 ± 1.36 b	n.d.	n.d.	11.42 ± 4.37 a	0.93 ± 0.55 c	9.60 ± 2.68 a	12.37 ± 3.66 a	n.d.
**23**	1150	2-(5-ethenyl-5-methyloxolan-2-yl)propanal [lilac aldehyde C]	2.40 ± 0.67	n.d.	n.d.	0.70 ± 0.54	n.d.	4.65 ± 3.08	2.11 ± 1.02	n.d.
**24**	1154	4-methyl-2-(2-methylprop-1-enyl)-3,6-dihydro-2H-pyran [nerol oxide]	6.51 ± 1.88 a	n.d.	0.42 ± 0.30 b	n.d.	n.d.	n.d.	n.d.	n.d.
**25**	1167	2-(5-ethenyl-5-methyloxolan-2-yl)propan-2-ol isomer I [epoxylinalool 1]	37.83 ± 4.86 a	n.d.	1.26 ± 0.62 b	6.00 ± 1.81 b	n.d.	9.28 ± 4.50 b	n.d.	n.d.
**26**	1173	**2-(5-ethenyl-5-methyloxolan-2-yl)propan-2-ol isomer II [epoxylinalool 2]**	23.86 ± 3.47 b	1.71 ± 0.52 b	2.62 ± 0.91 b	10.33 ± 3.03 b	1.10 ± 0.41 b	50.15 ± 20.35 a	2.81 ± 0.88 b	n.d.
**27**	1184	butanedioic acid [succinic acid]	4.91 ± 2.42	16.29 ± 7.76	8.39 ± 6.02	n.d.	n.d.	n.d.	n.d.	n.d.
**28**	1188	2-(4-methyl-1-cyclohex-3-enyl)propan-2-ol [terpineol]	2.07 ± 0.66	n.d.	n.d.	n.d.	n.d.	n.d.	n.d.	n.d.
**29**	1196	2,6,6-trimethylcyclohexa-1,3-diene-1-carbaldehyde [safranal]	n.d.	n.d.	n.d.	1.56 ± 0.75	n.d.	9.29 ± 7.06	n.d.	n.d.
**30**	1199	tetrahydro-β,5-dimethyl-5-vinyl-2-furanethanol isomer I [lilac alcohol A]	2.50 ± 0.71	n.d.	2.60 ± 1.38	n.d.	n.d.	n.d.	n.d.	n.d.
**31**	1201	tetrahydro-β,5-dimethyl-5-vinyl-2-furanethanol isomer III [lilac alcohol B]	4.98 1.45	2.36 ± 0.75	n.d.	n.d.	n.d.	n.d.	n.d.	n.d.
**32**	1208	tetrahydro-β,5-dimethyl-5-vinyl-2-furanethanol isomer II [lilac alcohol C]	1.59 ± 0.46	n.d.	n.d.	n.d.	n.d.	n.d.	n.d.	n.d.
**33**	1226	**methyl nonanoate**	9.65 ± 2.14	n.d.	2.09 ± 0.97	14.76 ± 5.07	1.80 ± 1.17	12.50 ± 9.31	n.d.	6.26 ± 3.30
**34**	1228	5-(hydroxymethyl)-2-furaldehyde [5-hydroxymethylfurfural]	n.d.	n.d.	1.93 ± 0.97	n.d.	n.d.	n.d.	n.d.	n.d.
**35**	1232	2,6,6-trimethyl-2-cyclohexen-1-ol	n.d.	n.d.	n.d.	1.12 ± 0.57	n.d.	n.d.	n.d.	n.d.
**36**	1233	2-hydroxy-3,5,5-trimethyl-2-cyclohexen-1,4-dione	5.81 ± 1.00 a	1.86 ± 0.18 b	n.d.	n.d.	n.d.	5.97 ± 2.85 a	n.d.	n.d.
**37**	1244	ethyl 2-phenylacetate	1.22 ± 0.55	0.87 ± 0.39	1.67 ± 0.93	n.d.	n.d.	n.d.	1.07 ± 0.67	n.d.
**38**	1250	4-methoxybenzaldehyde [anisaldehyde]	n.d.	n.d.	n.d.	n.d.	n.d.	n.d.	1.90 ± 1.09	n.d.
**39**	1253	tetrahydro-β,5-dimethyl-5-vinyl-2-furanethanol isomer IV [lilac alcohol D]	1.61 ± 0.52	n.d.	n.d.	n.d.	n.d.	n.d.	n.d.	n.d.
**40**	1257	2-phenylethyl acetate	1.48 ± 1.24	3.79 ± 1.35	7.86 ± 7.07	n.d.	n.d.	n.d.	n.d.	n.d.
**41**	1280	(4-methoxyphenyl)methanol [anise alcohol]	n.d.	n.d.	n.d.	n.d.	n.d.	n.d.	1.31 ± 1.03	n.d.
**42**	1297	ethyl nonanate	3.33 ± 1.62	3.81 ± 1.19	2.91 ± 1.85	n.d.	n.d.	n.d.	n.d.	n.d.
**43**	1314	3,4,5-trimethyl-phenol	n.d.	n.d.	n.d.	n.d.	n.d.	n.d.	5.69 ± 2.09	n.d.
**44**	1325	methyl decanoate	1.59 ± 0.47	n.d.	0.64 ± 0.33	1.94 ± 0.71	n.d.	n.d.	0.52 ± 0.33	1.08 ± 0.64
**45**	1329	2,2-dimethyl butanal	3.94 ± 1.45	n.d.	n.d.	n.d.	n.d.	n.d.	n.d.	n.d.
**46**	1382	(*E*)-1-(2,6,6-trimethylcyclohexa-1,3-dien-1-yl)but-2-en-1-one [damascenone]	6.96 ± 1.16 a	1.49 ± 0.88 b	2.15 ± 1.07 ab	0.47 ± 0.36 b	n.d.	0.63 ± 0.36 b	n.d.	n.d.
**47**	1389	diethyl hexanedioate	n.d.	0.41 ± 0.25	n.d.	n.d.	n.d.	n.d.	n.d.	n.d.
**48**	1396	ethyl decanoate	0.40 ± 0.23 b	1.28 ± 0.50 a	1.57 ± 0.80 ab	n.d.	n.d.	n.d.	n.d.	n.d.
**49**	1400	tetradecane	0.28 ± 0.20	n.d.	n.d.	n.d.	n.d.	n.d.	n.d.	n.d.
**50**	1500	pentadecane	1.25 ± 0.77	n.d.	n.d.	n.d.	n.d.	n.d.	n.d.	n.d.
**51**	1516	(*Z*)-5,6-dimethyl-1,3-cyclohexadiene	n.d.	n.d.	n.d.	n.d.	n.d.	2.18 ± 0.80	n.d.	n.d.
**52**	1537	methyl 10-oxodecanoate	n.d.	n.d.	n.d.	0.39 ± 0.26	n.d.	n.d.	n.d.	n.d.
**53**	1597	ethyl dodecanoate	n.d.	2.59 ± 1.26	1.60 ± 1.27	n.d.	n.d.	n.d.	n.d.	n.d.
**54**	1600	hexadecane	0.46 ± 0.27	n.d.	n.d.	n.d.	n.d.	n.d.	n.d.	n.d.
**55**	1646	1,4-dimethylindanyl acetate	8.11 ± 1.62 a	n.d.	n.d.	0.90 ± 0.61 b	n.d.	n.d.	n.d.	n.d.
**56**	1652	dimethyl decanedioate	1.66 ± 0.48 ab	n.d.	0.41 ± 0.27 b	7.07 ± 1.85 a	1.56 ± 0.82 ab	1.53 ± 0.52 ab	n.d.	4.09 ± 3.54 ab
**57**	1727	methyl tetradecanoate	n.d.	0.61 ± 0.38	n.d.	0.36 ± 0.25	1.56 ± 0.71	n.d.	n.d.	n.d.
**58**	1790	diethyl decanedioate	n.d.	1.08 ± 0.81	n.d.	n.d.	n.d.	n.d.	n.d.	n.d.
**59**	1796	ethyl tetradecanoate	n.d.	4.04 ± 1.59	1.14 ± 0.66	n.d.	n.d.	n.d.	n.d.	n.d.
**60**	1922	metyl hexadecanoate	0.66 ± 0.19	2.36 ± 0.73	2.22 ± 0.61	2.40 ± 1.23	3.59 ± 0.96	0.55 ± 0.32	5.60 ± 4.01	n.d.
**61**	1977	ethyl hexadecanoate	n.d.	9.30 ± 2.84	5.90 ± 4.23	n.d.	n.d.	n.d.	1.60 ± 1.03	n.d.
**62**	2051	methyl oleate	0.77 ± 0.21	n.d.	n.d.	0.36 ± 0.18	n.d.	n.d.	n.d.	n.d.

Values with different letters in the columns indicate statistical difference at P ≤ 0.05. nd: not detected

LRI: calculated linear retention index

*Common names of the VOCs are present in the square brackets. 10 dominant volatiles are present in bold.

Lilac alcohol B was only found in longan honey (both *A*. *mellifera* and *A*. *cerana*) (Table 2). Furthermore, 1,5,8-p-menthatriene, ethyl heptanoate, terpineol, lilac alcohol C, lilac alcohol D, 2,2-dimethyl butanal, tetradecane, pentadecane and hexadecane were only found in longan honey from *A*. *mellifera* (Table 2). Diethyl hexanedioate and diethyl decanedioate were only found in longan honey from *A*. *cerana*. Methyl caproate, benzaldehyde and 5-hydroxymethylfurfural were only found in wild honey from *A*. *dorsata* while methyl 10-oxodecanoate, 2,6,6-trimethyl-2-cyclohexen-1-ol and methyl oleate were only found in wild honey from *A*. *mellifera*. *(Z)*-5,6-dimethyl-1,3-cyclohexadiene and were only found in lychee honey. 2-Furanmethanol, butyryl lactone, phenylmethanol, anisaldehyde, anise alcohol and 3,4,5-trimethyl-phenol were only found in coffee honey from *A*. *cerana* (Table 2). Interestingly, 2-hydroxy-3,5,5-trimethyl-2-cyclohexen-1,4-dione was only found in lychee from *A*. *mellifera* and longan honey from both *A*.*mellifera* and *A*. *cerana*.

**Table 2:** List of VOCs that could serve as possible floral markers and their odor descriptors.

	Honey		Compound	Odor descriptor
**Present**	longan honey (*A*. *mellifera*)	1	2-methyl-5-prop-1-en-2-ylcyclohexa-1,3-diene [1,5,8-*p*-menthatriene]	roasted
		2	ethyl heptanoate	fruity
		3	2-(4-methyl-1-cyclohex-3-enyl)propan-2-ol [terpineol]	odorless
		4	tetrahydro-β,5-dimethyl-5-vinyl-2-furanethanol isomer II [lilac alcohol C]	green, grassy and fresh
		5	tetrahydro-β,5-dimethyl-5-vinyl-2-furanethanol isomer IV [lilac alcohol D]	green, grassy and fresh
		6	2,2-dimethyl butanal	-
		7	hexadecane	-
		8	tetradecane	waxy
		9	pentadecane	waxy
	longan honey (*A*. *mellifera* & *A*. *cerana*)	10	tetrahydro-β,5-dimethyl-5-vinyl-2-furanethanol isomer III [lilac alcohol B]	green, grassy and fresh
	longan honey (*A*. *cerana*)	11	diethyl hexanedioate	-
		12	diethyl decanedioate	fruity
	wild honey (*A*. *dorsata*)	13	methyl caproate	fruity
		14	benzaldehyde	almond, fruity, powdery, nutty and benzaldehyde-like
		15	5-(hydroxymethyl)-2-furaldehyde [5-hydroxymethylfurfural]	fatty, buttery, musty, waxy and caramellic
	wild honey (*A*. *mellifera*)	16	methyl 10-oxodecanoate	-
		17	methyl oleate	fatty
		18	2,6,6-trimethyl-2-cyclohexen-1-ol	-
	lychee honey (*A*. *mellifera*)	19	(*Z*)-5,6-dimethyl-1,3-cyclohexadiene	-
	coffee honey (*A*. *cerana*)	20	2-furanmethanol	brown caramellic, bready and coffee
		21	butyryl lactone	Creamy, fatty and dairy-like
		22	phenylmethanol	floral
		23	4-methoxybenzaldehyde [anisaldehyde]	sweet, powdery, vanilla, anise and woody
		24	(4-methoxyphenyl)methanol [anise alcohol]	weet, powdery creamy, balsamic and coumarin
		25	3,4,5-trimethyl-phenol	green
**Absent**	longan honey (*A*. *cerana*)	26	2-phenylacetaldehyde	honey and floral rose
	Sunflower (*A*.*mellifera*)	27	3,7-dimethylocta-1,6-dien-3-ol (linalool)	citrus, orange, floral, terpy, waxy and rose
		28	(5E)-3,7-dimethylocta-1,5,7-trien-3-ol [ho-trienol]	sweet, tropica and ginger
		29	benzyl ethanol	Sweet, floral and fruity
		30	2-(5-ethenyl-5-methyloxolan-2-yl)propan-2-ol isomer II [epoxylinalool 2]	-
		31	metyl hexadecanoate	Waxy

Common names of the VOCs are present in the square brackets.

**Fig 2:**
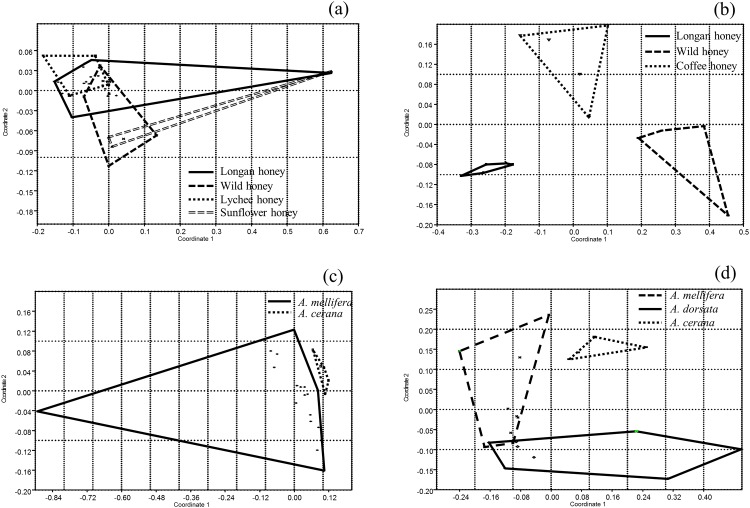
Groupings among volatile organic compounds of (a) longan honey, wild honey, lychee honey and sunflower honey from *A*. *mellifera* (b) longan honey, wild honey, coffee honey from *A*. *cerana* showing how VOCs from same honeybee species are clustered or separated from VOCs from different floral sources; (c) longan honey from *A*. *mellifera* and *A*. *cerana* (d) wild honey from *A*. *mellifera*, *A*. *dorsata* and *A*. *cerana* showing how VOCs from same floral sources are clustered or separated from VOCs from different honeybee species.

Non-metric multidimensional scaling and plot grouping analysis revealed that differences in floral sources and honeybee species lead to differences in VOC groupings. Among volatiles from honey produced by the same honeybee species, *A*. *mellifera*, longan honey was the largest group with numerous volatile compounds (Fig 2a). Volatiles in longan honey slightly overlapped with volatiles from three different groups of floral sources namely, lychee, wild and sunflower (Fig 2a). Likewise, the volatile group of wild honey overlapped with the longan, lychee and sunflower groups. However, volatiles belonging to lychee honey were completely separated from volatiles belonging to sunflower honey (Fig 2a). There were clear differences among volatiles of honey produced by *A*. *cerana* from three different floral sources (longan, wild and coffee) (Fig 2b). Volatiles from longan honey produced by *A*. *mellifera* and *A*. *cerana* were distinctly different (Fig 2c). Moreover, volatiles from wild honey produced by *A*. *cerana* was distinctly different from those produced by *A*. *mellifera* and *A*. *dorsata* (Fig 2d) but wild honey from *A*. *mellifera* and *A*. *dorsata* slightly overlapped (Fig 2d). These results reflect the differences among VOC groups due to floral sources and honeybee species.

## Discussion

This study is the first to investigate the volatile organic compounds of Thai honeys from several floral sources and different honeybee species. In Thailand, honey is often marketed as unifloral honey. The floral origin of the honey gives it a unique flavor and fragrance. In fact, it is not only the botanical origin of flora that influence the quality of honey but also some extended factors such as honeybee species and storage condition. This present study focused on the influence of floral source and honeybee species on aroma of honey.

To the best of our knowledge, butyryl lactone, 1,8-nonadiyne, ethyl heptanoate, 2,6,6-trimethyl-2-cyclohexen-1-ol, 2-hydroxy-3,5,5-trimethyl-2-cyclohexen-1,4-dione, anise alcohol, 2,2-dimethyl butanal and *(Z)*-5,6-dimethyl-1,3-cyclohexadiene, which were found in Thai honeys in this study have never been reported in other honeys. Moreover, some particular volatiles in the headspace of specific honey types have been considered to be floral markers for those particular honeys. Thirty-one of such potential markers were observed. For instance, 1,5,8-p-menthatriene, ethyl heptanoate, terpineol, lilac alcohol C, lilac alcohol D, 2,2-dimethyl butanal, pentadecane and hexadecane were classified as markers for longan honey from *A*. *mellifera* while diethyl hexanedioate and diethyl decanedioate were classified as markers for longan honey from *A*. *cerana*. Furthermore, lilac alcohol B was classified as a marker for longan honey from both *A*. *mellifera* and *A*. *cerana*. Methyl caproate, benzaldehyde and 5-hydroxymethylfurfural were classified as markers for wild honey from *A*. *dorsata* while methyl 10-oxodecanoate, 2,6,6-trimethyl-2-cyclohexen-1-ol and methyl oleate were classified as markers for wild honey from *A*. *mellifera*. (*Z*)-5,6-dimethyl-1,3-cyclohexadiene could be characteristic volatiles for lychee honey from *A*. *mellifera* whiles 2-furanmethanol, butyryl lactone, phenylmethanol, anisaldehyde, anise alcohol and 3,4,5-trimethyl-phenol could be considered as markers for coffee honey from *A*. *cerana*. Although, these identified volatiles were detected in small quantities they could be considered as marker VOCs because of their specificity and/or uniqueness to those honeys. On the contrary, although the amounts of *cis-* and *trans-*linalool oxide were notably high, they could not be considered specific to a particular honey because they have been found in both lychee honey (*A*. *mellifera*) and longan honeys (*A*.*mellifera* and *A*. *cerana*). However, 2-hydroxy-3,5,5-trimethyl-2-cyclohexen-1,4-dione was found in longan and lychee honeys which could be because of these two honey originated from floral sources of the same family (Sapindaceae) [19].

An important finding of this study is that volatiles emitted by honeys are dependent on both the floral source and honeybee species that produced the honey. Honeybees feeding on different floral sources produce honeys with different quality and quantity of volatiles. The highest number of volatiles detected were from longan honey followed by wild honey, lychee honey, coffee honey and sunflower honey, which had the least number of volatiles. This finding supports the evidence that longan honey is the most fragrant honey, while sunflower honey is the least fragrant honey among monofloral honey as highlighted by Thai beekeepers. In addition, this is in agreement with many previous studies that found floral sources to be a major factor for differentiating honey aroma [4, 7, 20, 21]. It is well known that wild honey is multifloral honey derived from a variety of nectar sources. It is therefore reasonable to suggest that VOCs of wild honey mimic a combination of VOCs from various monofloral honeys. This is in agreement with our results, which showed that the group of VOCs from wild honey overlapped with every other group of monofloral VOCs. In particular, plants from same family tended to have similar quality and quantity of volatile compounds. It was therefore not surprising that longan and lychee both of which belong to the family Sapindaceae [19] had groups of volatiles that overlapped as seen in Fig 2a. As expected, the VOCs of sunflower (family: Asteraceae) [22], barely overlapped with other monofloral groups.

So far, relatively few studies have focused on the honeybee species as a factor that causes differences in honey aroma [23, 24]. As it is depicted in Fig 2, showing volatile organic compounds plot grouping of honey produced from different floral sources and bee species. The volatile compounds of honey produced by *A*. *mellifera* were overlapped (Fig 2a). On the contrary, as shown in Fig 2b, volatile compounds of honey produced by *A*. *cerana* were clearly grouped. Additionally, Fig 2c and 2d confirm that bee species affect the volatiles of honey even if they feed on the same floral source even though a slight overlap of VOCs was observed between wild honey from *A*. *mellifera* and *A*. *dorsata*. This study put the spotlight on the honeybee as an important factor that could influence the aroma of honey. The differences in the volatile profiles of the different monofloral honeys are likely influenced by the different honeybee species. For instance, we found that 2-phenylacetaldehyde was absent only in longan honey produced by *A*. *cerana*. Meanwhile longan honey from *A*. *mellifera* produced more volatiles than any other honey. The foraging behavior of bees that keeps nectar in their honey stomach for some time during their flight back to hives before transferring them into honey combs contribute to these observed differences. During the period that nectar is in the honey stomach, they may be transformed and that likely influence the VOCs. A recent study (18) in which a unique lactic acid bacteria (LAB) symbiont was discovered gives credence to this assertion. In that study, it was established that symbionts from the honey stomach of honeybees influence the production of metabolites including volatile compounds. Most importantly, the LAB symbionts in the honeybees’ honey stomach have also been found to influence antimicrobial properties of tested honeys. Furthermore, the lactic acid bacteria vary in different honeybee species [25]. Therefore, it may be possible that the metabolites produced due to LAB symbionts from different honeybee species would have impact not only on antibacterial properties but could also vary the volatile compounds in honeys. Foraging distance in each honeybee species could also be a factor that contributed to varied volatile compounds in tested honeys. It was found that in wild honey collected from *A*. *dorsata* provided more volatiles than ones from *A*. *mellifera* and *A*. *cerana*. It is known that *A*. *dorsata* can forage beyond 5 km [26], *A*. *mellifera* may forage up to 3 km [27] while *A*. *cerana* around 1–1.5 km [27]. The longer foraging distance may increase the chance to collect nectar from various sources leading to more detected volatile compounds in tested honeys. However, more investigation is required.

Many of the dominant volatiles in this study have been reported as common honey volatiles e.g. *cis*-linalool oxide [28–30], *trans*-linalool oxide [28, 30–35], ho-trienol [28, 30, 36, 37], epoxylinalool [38], 2-phenylacetaldehyde [4, 8, 39–41], furan-2,5-dicarbaldehyde [42], benzyl ethanol [7, 42, 43], isophorone [21, 44, 45] and methyl nonanoate [46, 47]. Above all, the identified compounds appeared to be typical constituents of Thai honey at various proportions and therefore provide the uniqueness of their aroma. Aside influencing the aroma of honey, the type and proportion of volatiles in the honey is known to influence the therapeutic properties including antioxidant, antibacterial and even immune enhancement. For instance, linalool and nerol oxide have been described as antibiotic volatiles which show broad-spectrum antimicrobial activity [48–57]. Moreover, linalool had previously been identified as an antioxidant volatile [58, 59], likewise safranal [60, 61], 4-oxoisophorone [62], and damascenone [62]. The antioxidant capacity of 4-oxoisophorone and damascenone have been found to have the potential to enhance the vertebrate immune system [63]. Based on the concentration of these compounds in the headspace of the honey samples, their biomedical ability could be slightly low compared to four salient factors of honey (hydrogen peroxide, high osmotic pressure, acidity and non-peroxide factors). It would be interesting to conduct further experiments to find out whether there are differences among volatiles of honey produced by the same bee species feeding on the same floral source in different geographical regions.

## Conclusion

There were a total of 63 compounds detected in the headspace of the honey samples. The highest (48) and the least (8) numbers of volatiles were identified in longan honey and sunflower honey, respectively. Each of the Thai honey samples studied had a complex and unique volatile organic compound profile which varied in quality and quantity from other honey samples. The variation in volatile compounds in the honey samples lead to uniqueness of fragrances, tastes and potential biomedical properties. These characteristic depend not only on the nectar-providing plant species, but also on honeybee species. Therefore, volatile compound profiles may be used as chemical markers for tracing the origin of honey. Additionally, NMDS and grouping plot can be used for controlling honey produced by different floral sources and bee species.
